# Dr. Vaughan, on Malformation

**Published:** 1800-06

**Authors:** W. Vaughan

**Affiliations:** Rochester


### *To the Editors of the Medical and Phyſical Journal.*

Gentlemen,

The following Hiſtory of a Mal-formation is, I think, worthy of a place in your Journal. In the firſt place, it is no common mal-formation; it is, on the contrary, as Dr. Baillie remarks, a very uncommon one. (Morbid Anatomy, page 181).

---

ſphincter; but where this is to be done nearly to the extent of the fore finger, as will be neceſſary in ſome caſes, a ſtraight knife will, without great care, be apt to cut the operator's finger. It may alſo be made of any degree of curvature, or with a handle, of any ſhape or ſize, inſtead of the ring.

181) Again, it ſuggeſts a very uſeful caution againſt truſting implicitly, on all occaſions, to the ſenſes. And laſtly, it proves, perhaps, that perſons of experience may, by that very experience, be liable, in ſome inſtances, to draw haſty and falſe concluſions. You will judge of theſe reflections; and you will do with them, and with my hiſtory, as may, in your opinion, beſt anſwer the purpoſes of your Journal. I am,

Gentlemen,

Your obedient ſervant,

W. VAUGHAN.

*Rocheſter,*

*January,* 20, 1800.

---

A few years ago, a child without an *anus* was born at Gillingham, near Rocheſter; and I was requeſted to ſee it. There was indeed no *anus*, nor even the leaſt index of one; but the *fæces* were voided from a concial body extending upwards, from under the *ſymphyſis pubis*, and reaching the umbilical region. The child ſucked ſtrongly; and the *fæces*, in an almoſt fluid ſtate, were diſcharged as often as the child cried.

The urine flowed, I obſerved it twice, from a part concealed by the baſe of the conical body.

Some pronounced the child a female; and others, thinking it a male, were deſirous to make an *anus*.

The child lived only a few days. When the cavity of the *abdomen* was expoſed, and the inteſtines traced as far as the laſt *vertebra* of the loins, it was found, that the *rectum*, inſtead of deſcending along the *os ſacrum*, paſſed immediately into the *vagina*. The prolapſed part, which was the *rectum* inverted, was eaſily drawn backwards, through the opening, into the *vagina*.

Now, is it not likely at leaſt, that the perſons who miſtook the protruded inteſtine for a *penis*, imagined, from their experience of Nature's monſtroſities, that the want of a *præputium*, and the preſence of a villous coat, were both *luſus naturæ?* If this were not the caſe, it is hard to account at all for their miſtake. This much is, however, certain, not only that the *re[illegible]um* was miſtaken for a *penis*, but alſo, that the *labia pudendi* were miſtaken for a *ſcrotum*, into which the *teſticuli* had not yet deſcended. If any attempt to form an *anus* had been made, the diſappointment of the operator muſt have been inconceivable and diſgraceful!

*Appendix*

